# Primary Stability of Implants Inserted Using Different Osseodensification Systems in Low-Density Bone: An In Vitro Study

**DOI:** 10.3390/dj14040214

**Published:** 2026-04-07

**Authors:** André Luís Onodera, Alexandre Wanderley Alécio, Gustavo Batista Grolli Klein, Sheila Cortelli, Rogério de Lima Romeiro

**Affiliations:** 1 Implant Dentistry, São Leopoldo Mandic School of Dentistry, Campinas 13045-755, SP, Brazilgutoklein@yahoo.com.br (G.B.G.K.);; 2 Implant Dentistry, School of Dentistry, University of Taubaté, Taubaté 12020-340, SP, Brazil

**Keywords:** dental implants, primary stability, bone density, osseointegration, torque, resonance frequency analysis, in vitro study

## Abstract

**Background/Objectives**: It remains unclear which drilling strategy is most effective for maximizing mechanical stability in low-density bone and whether high insertion torque is determinative. The aim of this in vitro study was to compare the primary stability of implants placed using different drilling protocols—conventional (CV), undersized (US), and osseodensification (OD). Three osseodensification systems—Versah burs (V), Bone Reamer Drills (WF), and Master Conical Densifiers (DSP)—were also compared. **Methods**: A set of 11 blocks was used for the drilling protocol comparison (CV, US, OD) and a separate set of 11 blocks was used for the osseodensification system comparison (V, WF, DSP). External-hexagon implants were inserted epicrestally. Insertion torque was measured using a torque meter, and implant stability quotients (ISQs) were assessed through resonance frequency analysis. **Results**: ISQ for OD was significantly higher than that for CV but statistically similar to that for US, whereas insertion torque for OD was significantly higher than that for both US and CV. A weak correlation was found between variables for CV and US, and a moderate one was observed for OD. Both WF and DSP showed significantly higher ISQ values than V. Insertion torque for DSP was significantly higher than that for both WF and V. A moderate correlation was found between variables for DSP and V, and a weak one for WF. **Conclusions**: In this invitro study, the OD protocol performed better than CV in terms of ISQ and better than both CV and US in terms of insertion torque. WF and DSP outperformed V in ISQ, whereas DSP yielded the highest insertion torque. Weak-to-moderate correlations between variables in both analyses indicated that higher insertion torque did not necessarily translate into greater stability.

## 1. Introduction

Successful osseointegration of dental implants has been linked to their primary stability. Nevertheless, recent systematic reviews indicate that low primary stability may not compromise implant survival when delayed loading protocols are used, since implants with implant stability quotient (ISQ) values < 60 or insertion torque values < 35 can achieve survival rates exceeding 95%. In fact, a significant relationship between primary stability and success has been primarily observed in studies on immediate loading.

Various implant systems and surgical protocols have been developed to optimize stability according to bone quality [1]. The surgical technique used for implant site preparation appears to have a decisive influence on stability outcomes, particularly in the posterior maxilla, where bone density is generally low. In this region, low insertion torque values are associated with reduced primary stability, which may lead to excessive micromotion and, as a result, implant failure [2,3]. The literature describes a critical micromotion threshold between 50 and 150 µm, above which fibrous encapsulation may occur, precluding stable osseointegration [3].

Although primary implant stability is not always recognized as a prerequisite for predictable osseointegration and long-term implant success, adequate mechanical engagement between the implant and surrounding bone minimizes displacement during the early healing phase and facilitates the establishment of direct bone-to-implant contact. Conversely, insufficient stability may result in excessive micromovement and compromised integration. For this reason, several surgical approaches, including undersized osteotomy and currently available osseodensification techniques, have been proposed to enhance mechanical engagement in low-density bone and improve initial implant fixation while differing in drill geometry and design [4].

In type III and IV bone, two main surgical approaches have been used to enhance bone–implant contact: undersized drilling and bone condensation [5]. The former uses a final drill diameter smaller than the implant diameter, whereas the latter uses condensers to laterally compact the trabecular bone, thereby increasing local density. Traditionally, implant site preparation is performed with subtractive drilling systems, using twist drills of progressively larger diameters at 800–1500 rpm under copious irrigation to prevent thermal injury to the bone [6,7,8].

More recently, non-subtractive drilling techniques have been proposed to compact rather than remove bone tissue [6]. The undersized osteotomy technique—achieved by omitting the final drill—creates an osteotomy slightly narrower than the implant diameter, thereby increasing insertion torque and reportedly improving primary stability [7,8,9]. However, excessive undersizing (>10%) may cause cortical compression, local ischemia, and microcracks, potentially resulting in delayed healing and early marginal bone loss [4,10].

Advances in mechanical engineering have led to the development of osseodensification drills designed to perform additive drilling by compacting trabecular bone laterally and apically, thereby increasing local density and improving mechanical interlocking between the bone and implant [11]. In addition, the autogenous bone particles compacted during the procedure may act as osteogenic mediators, promoting faster bone healing and secondary stability [12]. Densifying burs feature a tapered geometry and specially designed flutes that allow both clockwise (drilling) and counterclockwise (densifying) motion, typically operating at 1100 rpm under continuous saline irrigation [13].

Currently, the most widely accepted biomechanical parameters for assessing implant primary stability are insertion torque (N·cm) and the ISQ, the latter measured by resonance frequency analysis (RFA) [14]. Insertion torque quantifies the mechanical resistance encountered during apical advancement of the implant, whereas the ISQ reflects the rigidity of the bone–implant interface and its susceptibility to micromovement [14]. The ISQ scale ranges from 0 to 100 (3500–85,000 Hz) and is commonly categorized as low (<60), moderate (60–70), or high (>70) stability [15].

Primary stability is closely related to bone density, which can be enhanced through osseodensification or undersized preparation compared with conventional drilling. Therefore, the aim of this study was to compare the primary stability of implants placed in low-density artificial bone using different drilling protocols—conventional (CV), undersized (US), and osseodensification (OD)—based on insertion torque and ISQ values. Additionally, three osseodensification systems—Versah burs (V), Bone Reamer Drills (WF), and Master Conical Densifiers (DSP)—were compared using the same parameters. The null hypothesis was that there would be no significant differences regarding insertion torque or primary implant stability among the drilling protocols and osseodensification systems evaluated.

## 2. Materials and Methods

Eleven polyurethane blocks measuring 14 cm × 2.5 cm × 2.5 cm and simulating type IV bone (Nacional Ossos; Jaú, SP, Brazil) were used for the drilling protocol comparison, and another 11 for the osseodensification system comparison. Each block was divided into three horizontal rows using a 2HB black pencil, with each row containing five compartments of 7 mm^2^, thereby providing five repetitions for each drilling protocol or osseodensification system within every block. A minimum distance of 3 mm was maintained between adjacent perforations.

The CV drilling protocol was performed using a drill kit with progressive diameters of 1.6, 2.0, 2.5, and 2.8 mm (Senior Surgical Kit; DSP Biomedical, Campo Largo, PR, Brazil); the US protocol used the same kit with drill diameters of 1.6, 2.0, and 2.5 mm; and the OD protocol used the Master Conical Densifiers kit (DSP Biomedical, Campo Largo, PR, Brazil) with drill diameters of 1.6, 2.0, 2.3, 2.5, and 3.5 mm.

**Figure 1:**
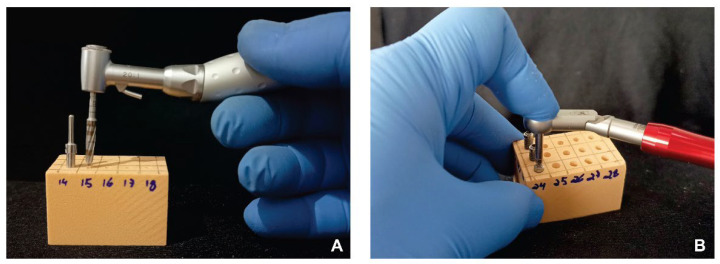
(**A**) Simulated osteotomy performed on a low-density artificial bone block using one of the tested drilling protocols. (**B**) Implant insertion and measurement of the corresponding insertion torque.

The tested osseodensification systems were applied using drill sequences of 2.0, 2.3, 2.5, 3.0, and 3.3 mm for the V system using Densah Drills (Versah Brasil, Joinville, SC, Brazil); 1.8, 2.2, 2.6, 3.0, and 3.4 mm for the WF system using Bone Reamer Drills (WF Cirúrgicos, São Paulo, SP, Brazil); and 1.6, 2.0, 2.3, 2.5, and 3.5 mm for the DSP system (DSP Biomedical, Campo Largo, PR, Brazil). The same BLM 600 PLUS motor (Driller, Carapicuíba, SP, Brazil) and 20:1 Push Button contra-angle (Driller) were used to perform all simulated osteotomies (Figure 1A).

In the blocks subjected to the drilling protocols, CV and US perforations were performed at 1200 rpm in a clockwise direction, and OD perforations at 800 rpm, also clockwise, as recommended by their respective manufacturers. In the blocks used for comparing the osseodensification systems, V and WF perforations were performed at 800 rpm in a counterclockwise direction, and DSP perforations at 800 rpm clockwise, also as recommended by their respective manufacturers. All perforations were performed to a depth of 11.5 mm. Of the 165 perforations planned for each comparison (11 blocks × 3 rows × 5 repetitions), 3 and 6 perforations were discarded in the drilling protocol and osseodensification system comparisons, respectively, due to compartment fracture during the simulated osteotomies, resulting in a total of 162 and 159 perforations. All perforations were performed freehand, without the use of a surgical guide, by the same operator to maintain consistency throughout the procedures.

**Figure 2:**
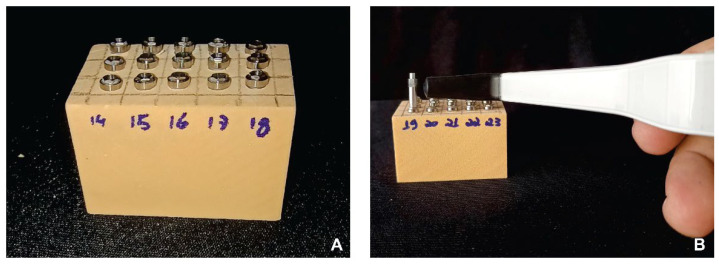
(**A**) Low-density artificial bone block with implants placed using the three tested drilling protocols. (**B**) Measurement of the implant stability quotient (ISQ) using the resonance frequency analysis (RFA) device.

After drilling, external hexagon implants (DSP Biomedical, Campo Largo, PR, Brazil) measuring 3.5 × 11.5 mm were installed in all blocks using a digital wrench (DSP Biomedical) until manual locking was achieved. A mechanical torque meter (DSP Biomedical) was then used to complete implant insertion to the level of the synthetic bone block (epicrestally). Insertion torque (N·cm) was measured using this mechanical torque meter (Figure 1B), and ISQ was assessed using a resonance frequency analysis system (Osstell AB, Gothenburg, Sweden; Figure 2A,B). For ISQ measurement, the SmartPeg (standard model, Osstell AB, Gothenburg, Sweden) was attached to the implant with the system’s SmartMount and digitally tightened until locking. The Osstell Beacon (Osstell AB, Gothenburg, Sweden) device was subsequently positioned near the top of the SmartPeg, without contact, at an angle of approximately 90°, avoiding the generation of magnetic pulses of different frequencies. Implant stability was then recorded as the implant stability quotient (ISQ).

### Statistical Analysis

The Friedman test was used to compare the ISQ and insertion torque values observed for the different drilling protocols and the different osseodensification systems. The Wilcoxon test was used for pairwise comparisons of the values assumed by these variables, and Spearman’s correlation test was used to assess potential correlations between the variables for each of the drilling protocols and osseodensification systems tested. Correlation coefficients (r) from |0.10| to |0.40| were considered indicative of a weak correlation; from |0.40| to |0.60|, a moderate correlation; and from |0.60| to |1.00|, a strong correlation. The analyses were performed using SPSS v. 26 (IBM, Armonk, NY, USA) and Minitab v. 21.2 (Minitab, State College, PA, USA) software. The significance level adopted was 5% and the confidence interval was 95%.

## 3. Results

Insertion torque and ISQ values for the drilling protocols and osseodensification systems are presented in Table 1 and Table 2, respectively. Significant differences were observed among the drilling protocols for both variables (*p* < 0.05, Table 1). Pairwise comparisons showed that OD yielded significantly higher ISQ values than CV (*p* < 0.05), whereas ISQ did not differ significantly between OD and US (*p* > 0.05, Table 3). OD also produced significantly higher insertion torque values than both CV and US, and US produced significantly higher insertion torque values than CV (*p* < 0.05, Table 3). The Spearman correlation test indicated a weak association between ISQ and insertion torque for CV and US, and a moderate association for OD (Table 4), suggesting that increases in insertion torque did not necessarily correspond to greater primary stability.

Significant differences were also found among the osseodensification systems for both variables (*p* < 0.05, Table 2). Pairwise comparisons showed that both WF and DSP yielded significantly higher ISQ values than V (*p* < 0.05), whereas no significant difference in ISQ was observed between WF and DSP (*p* > 0.05, Table 3). DSP produced significantly higher insertion torque values than both WF and V (*p* < 0.05), and WF produced significantly higher insertion torque values than V (*p* < 0.05, Table 3). The Spearman correlation analysis revealed a moderate association between ISQ and insertion torque for DSP and V, and a weak association for WF (Table 4).

## 4. Discussion

The relationship between insertion torque and implant stability is more complex than a simple mechanical correlation. Although increased torque is often interpreted as an indicator of improved primary fixation, excessive compression of peri-implant bone may have relevant biological consequences during the early healing phase. Excessive mechanical compression of trabecular bone may reduce vascular spaces and compromise local blood supply. Reduced vascularization can induce microdamage, local ischemia, and delayed bone remodeling, potentially impairing the biological processes required for successful osseointegration [2,9]. Therefore, surgical techniques designed to enhance implant stability should aim to improve mechanical engagement while preserving bone vitality, particularly in low-density bone where trabecular architecture is more susceptible to vascular compromise.

The present in vitro study compared the primary stability associated with different drilling protocols and osseodensification systems in low-density artificial bone based on insertion torque and ISQ values. Higher insertion torque values were recorded for osseodensification drills, thereby rejecting the null hypothesis for this variable. However, these results did not correlate directly with higher ISQ, indicating that mechanical compression during implant insertion does not necessarily enhance implant–bone stiffness. This finding aligns with the latest clinical and experimental studies conducted by Moghaddas et al. [16] and Brunel et al. [17].

The former conducted an in vitro comparative analysis, while the latter performed a multicenter clinical trial involving 600 implants placed in soft bone. Both studies observed a weak-to-moderate relationship between insertion torque and ISQ despite improved densification, confirming the results of the present investigation. These findings suggest that the drilling technique plays a critical role in primary stability, consistent with previous reports by Antonacci et al. [7] and Oliveira et al. [13], but that insertion torque may not be a decisive factor. In our findings, the DSP osseodensification system produced the highest insertion torque, whereas both the WF and DSP burs yielded higher ISQ than V. Similar outcomes were reported by Barberá-Millán et al. [14] and Fernández-Olavarría et al. [18], who demonstrated that densifying drills enhance peri-implant bone density and contact. However, as in our study, they found that the mechanical gain in torque did not correspond to a proportional increase in resonance stability, supporting the non-linear relationship between torque and ISQ also identified by Degidi et al. [4] and Stocchero et al. [3].

Insertion torque has traditionally been used as an indicator of primary stability, but it merely reflects resistance to apical advancement, not rigidity of the implant–bone interface measured by RFA. Excessive torque can lead to over-compression, microfractures, or local ischemia, reducing osseointegration potential despite good initial fixation [2,9]. Our findings reinforce these concerns: the OD drilling protocol and DSP osseodensification system achieved greater insertion torque, but the correlation with ISQ remained weak to moderate.

Recent investigations by Moghaddas et al. [16] and Brunel et al. [17] provide clinical confirmation of this phenomenon, showing that implants placed in soft bone using osseodensification achieved higher insertion torque yet no statistically significant difference in ISQ compared with conventional drilling. These studies collectively indicate that increased torque reflects mechanical friction rather than enhanced biological integration.

Osseodensification promotes bone compaction instead of removal, increasing local density and potentially improving primary mechanical engagement. Oliveira et al. [13] demonstrated superior bone-to-implant contact and higher removal torque with densifying drills in animal models. However, the process is highly dependent on drill geometry, feed rate, and applied pressure, as noted by Slete et al. [12] and Comuzzi et al. [15]. The present findings support this interpretation, as differences between DSP, WF, and V results likely stemmed from specific flute designs and cutting angles. Thus, osseodensification should be regarded as a system-dependent mechanical process, whose success relies on instrumentation control and a balance between compaction and preservation of bone vitality.

Micromotion at the bone–implant interface represents a critical determinant of implant integration. Micromotion corresponds to minimal displacement of the implant relative to the surrounding bone and, when excessive, may interfere with the establishment of osseointegration by promoting fibrous tissue formation rather than direct bone-to-implant contact [3,19]. Experimental evidence suggests that micromotion should remain below approximately 150 µm to allow stable bone healing, whereas greater displacements may compromise implant fixation and increase the risk of failure. Consequently, implant site preparation should aim to achieve sufficient mechanical stability to limit micromotion while preserving bone physiology and vascularization, particularly in areas of low bone density.

Clinically, achieving adequate primary stability in low-density bone remains challenging. According to Tettamanti et al. [5] and Alghamdi & Jansen [6], insertion torques between 35–45 N·cm and ISQ values above 65 are considered favorable for early or immediate loading. In the present study, the OD protocol and DSP system reached these thresholds, suggesting that osseodensification may facilitate early loading protocols in compromised bone. Nevertheless, our results agree with the prospective clinical study of Abdelraouf et al. [20], who compared osseodensification and conventional drilling in the posterior maxilla and found that excessive compaction did not improve implant survival or marginal bone stability. This reinforces that stability assessment should rely primarily on RFA rather than torque alone, particularly when densifying burs are used in soft bone.

Among the limitations of the present in vitro study is the use of polyurethane blocks to simulate type IV bone. Although this model allows standardization and reproducibility, it does not replicate biological conditions such as vascularity, remodeling, and bone healing. Future studies should incorporate histomorphometric and fatigue analyses, as proposed by Tian et al. [11] and Delgado-Ruiz et al. [21], to assess how mechanical compression translates into biological osseointegration over time. Additionally, including three-dimensional micro-CT evaluation and in vivo histologic validation could clarify whether the torque–ISQ discrepancy persists after healing. Another limitation of the present study is that multiple osteotomies were performed within the same polyurethane block. Although a minimum distance of 3 mm was maintained between adjacent perforations to minimize interaction, the possibility of localized alterations in the material properties after repeated drilling cannot be completely excluded, which may introduce a degree of pseudoreplication. Additionally, differences in drilling direction among the evaluated osseodensification systems may have influenced the pattern and magnitude of bone compaction, potentially affecting the measured insertion torque and ISQ values. These factors should therefore be considered when interpreting the results, and future studies capable of addressing these concerns are also warranted. Yet another limitation of the present study is that a convenience sampling approach was adopted, as the available polyurethane blocks, drill kits, and measuring devices limited the scope. While this enabled preliminary exploratory analyses, it restricts the generalizability of the findings, and future studies should incorporate a calculated sample size to validate these results.

Overall, this study demonstrates that osseodensification drills increase insertion torque and may improve mechanical interlocking in low-density bone, but torque and ISQ describe distinct biomechanical phenomena—frictional resistance versus interfacial stiffness. The weak correlation between these parameters, corroborated by the most recent evidence, supports using both metrics to accurately assess implant stability. This in vitro study suggests that controlled osseodensification is beneficial for achieving adequate torque without jeopardizing bone physiology, especially in areas of low bone density.

## 5. Conclusions

• In this in vitro study, the OD protocol performed better than CV in terms of ISQ and better than both CV and US in terms of insertion torque.
• WF and DSP outperformed V in ISQ, whereas DSP yielded the highest insertion torque.
• Weak-to-moderate correlations between variables in both analyses indicated that higher insertion torque did not necessarily translate into greater stability.

## Figures and Tables

**Table 1:** Implant stability coefficient (ISQ) and insertion torque values for the drilling protocols tested.

	Drilling Protocol	Mean ± SD	Median	Q1	Q3	N	CI 95%	*p*-Value *
ISQ	CV	56.96 ± 1.76	57.0	56.0	58.0	54	0.47	0.004
	US	56.37 ± 4.02	58.0	55.0	59.0	54	1.07	
	OD	57.93 ± 2.08	58.0	57.0	59.8	54	0.56	
Insertion torque (N·cm)	CV	32.41 ± 6.35	30.0	30.0	30.0	54	1.69	<0.001
	US	43.00 ± 8.04	45.0	45.0	45.0	54	2.15	
	OD	54.07 ± 8.58	60.0	45.0	60.0	54	2.29	

CV = conventional drilling; US = undersized drilling; OD = osseodensification; SD = standard deviation; Q1 = first quartile; Q3 = third quartile; CI = confidence interval. * Friedman test (*p* < 0.05).

**Table 2:** Implant stability coefficient (ISQ) and insertion torque values for the osseodensification systems tested.

	Osseodensification System	Mean ± SD	Median	Q1	Q3	N	CI 95%	*p*-Value *
ISQ	V	57.66 ± 2.71	58.0	56.0	60.0	53	0.73	0.001
	WF	58.96 ± 2.27	59.0	58.0	60.0	53	0.61	
	DSP	58.70 ± 2.45	59.0	57.0	61.0	53	0.66	
Torque (N·cm)	V	28.40 ± 7.32	30.0	20.0	30.0	53	1.97	<0.001
	WF	32.55 ± 6.25	30.0	30.0	40.0	53	1.68	
	DSP	48.02 ± 6.75	50.0	45.0	50.0	53	1.82	

V = Densah burs; WF = Bone Reamer Drills; DSP = Master Conical Densifiers. SD = standard deviation; Q1 = first quartile; Q3 = third quartile; CI = confidence interval. * Friedman test (*p* < 0.05).

**Table 3:** Pairwise comparisons among drilling protocols and osseodensification systems tested regarding implant stability coefficient (ISQ) and insertion torque values.

Comparison	ISQ (*p-*Value *)	Insertion Torque (*p*-Value *)
CV × US	0.036	<0.001
CV × OD	0.002	<0.001
US × OD	0.870	<0.001
V × WF	<0.001	<0.001
V × DSP	0.027	<0.001
WF × DSP	0.372	<0.001

CV = conventional drilling; US = undersized drilling; OD = osseodensification; V = Densah burs; WF = Bone Reamer Drills; DSP = Master Conical Densifiers. * Wilcoxon test (*p* < 0.05).

**Table 4:** Correlation between implant stability coefficient (ISQ) and insertion torque for the drilling protocols and osseodensification systems tested.

		Correlation Coefficient (*r*) *	Interpretation
Drilling protocol	CV	0.222 (*p* = 0.106)	Weak
	US	–0.069 (*p* = 0.622)	Weak
	OD	0.385 (*p* = 0.004)	Moderate
Osseodensification system	V	0.317 (*p* = 0.022)	Moderate
	WF	0.231 (*p* = 0.096)	Weak
	DSP	0.325 (*p* = 0.018)	Moderate

CV = conventional drilling; US = undersized drilling; OD = osseodensification; V = Densah burs; WF = Bone Reamer Drills; DSP = Master Conical Densifiers. * Spearman correlation test (*p* < 0.05).

## Data Availability

The original contributions presented in this study are included in the article material. Further inquiries can be directed to the corresponding author.

## Abbreviations

The following abbreviations are used in this manuscript:

CV	conventional drilling protocol
US	undersized drilling protocol
OD	osseodensification drilling protocol
V	Versah burs osseodensification system
WF	Bone Reamer Drills osseodensification system
DSP	Master Conical Densifiers osseodensification system
ISQ	Implant stability quotient
RFA	Resonance frequency analysis
SD	Standard deviation
Q1	First quartile
Q3	Third quartile
CI	Confidence interval
